# A Comprehensive Review of Candidemia and Invasive Candidiasis in Adults: Focus on the Emerging Multidrug-Resistant Fungus *Candida auris*

**DOI:** 10.3390/diseases13040093

**Published:** 2025-03-24

**Authors:** Deobrat Chandra Mallick, Nayanjyoti Kaushik, Lokesh Goyal, Lipika Mallick, Prabhat Singh

**Affiliations:** 1 Christus Spohn Hospital Shoreline, 600 Elizabeth St, Corpus Christi, TX 78404, USA; lokesh0912@gmail.com; 2 CHI Health Nebraska Heart Institute, 7440 S 91st, Lincon, NE 68526, USA; kaushik_nayan@yahoo.co.in; 3 College of Arts and Science, Cornell University, Ithaca, NY 14850, USA; lmallick2005@gmail.com; 4 Kidney Specialist of South Texas, 1521 S Staple St, Corpus Christi, TX 78413, USA; drprabhatsingh@hotmail.com

**Keywords:** *Candida auris*, candidemia, invasive candidiasis, multidrug resistance, notifiable disease

## Abstract

Candidemia and invasive candidiasis represent critical healthcare-associated fungal infections that pose substantial challenges to medical systems worldwide. These conditions arise when fungi from the Candida genus infiltrate the bloodstream or deeper tissues, leading to a range of clinical manifestations. Among the various species, *Candida albicans* continues to hold its position as the most frequently encountered causative agent, largely due to its prevalence and adaptability within human hosts. However, it is far from the only significant player; other Candida species, such as *Candida glabrata*, *Candida parapsilosis*, and the particularly concerning *Candida auris*, contribute significantly to the disease burden and exhibit varying dominance depending on geographic regions. The clinical presentation of these infections can differ widely, spanning from subtle, almost imperceptible symptoms in some patients to severe, life-threatening fulminant sepsis in others, often accompanied by alarmingly high mortality rates that underscore the urgency of effective management strategies. Several well-established risk factors predispose individuals to developing invasive candidiasis and candidemia. Breaches in the body’s natural barriers—such as the skin (cutaneous) or the gastrointestinal (GI) tract—provide entry points for these opportunistic pathogens. Additionally, deficiencies in the host’s immune responses, whether due to medical treatments, underlying diseases, or genetic predispositions, heighten vulnerability to infection. Among the diverse Candida species, *Candida auris* has emerged as an especially troubling entity in recent years. This multidrug-resistant species is notorious for its resistance to standard antifungal therapies, which complicates treatment efforts and contributes to elevated morbidity and mortality rates. Its rapid global spread has positioned it as a formidable public health threat, prompting heightened surveillance and research into its behavior and control.

## 1. Methodology

To compile the information presented in this article, a comprehensive literature search was undertaken using multiple reputable academic databases, including PubMed, ScienceDirect, and Google Scholar. These platforms were selected for their extensive repositories of peer-reviewed scientific studies and their relevance to medical and microbiological research. The search strategy involved the use of specific key terms designed to capture the breadth of the topic: “candidemia”, “invasive candidiasis”, “*Candida auris*”, “multidrug-resistant fungus”, “symptoms”, “prevention”, and “treatment”. These terms were chosen to ensure a thorough exploration of the epidemiology, clinical features, and therapeutic approaches related to these infections. The retrieved studies were carefully reviewed by physician authors with expertise in infectious diseases, who evaluated the quality and relevance of the data. The findings were then synthesized and organized into this article, with an emphasis on clarity and accessibility to ensure that readers—whether healthcare professionals, researchers, or students—could readily understand and apply the information.

## 2. Introduction

The term “candidemia” specifically refers to the presence of Candida species circulating within the bloodstream, a condition that signals a serious breach of the body’s defenses. In contrast, “invasive candidiasis” encompasses a broader category of systemic infections caused by these fungi, which may or may not involve detectable candidemia. Examples of invasive candidiasis include hepatosplenic candidiasis (affecting the liver and spleen), *Candida* endocarditis (involving the heart valves), and infections of the central nervous system, each presenting unique diagnostic and therapeutic challenges. When Candida is identified in blood cultures, it is imperative that clinicians treat it as a significant finding rather than dismissing it as a mere contaminant. Such a discovery necessitates a meticulous investigation to pinpoint the source of the infection, which could range from indwelling medical devices to breaches in mucosal barriers. Importantly, simply removing the identified source—such as a contaminated catheter—is insufficient to resolve candidemia; antifungal therapy is an essential component of treatment and must always be administered to eradicate the infection [1].

Individuals with compromised immune systems, such as those undergoing chemotherapy or organ transplantation, and patients in intensive care units (ICUs) face the highest risk of developing these infections due to their weakened defenses and frequent exposure to invasive procedures. Historically, *Candida albicans* has been the predominant species responsible for these conditions, owing to its widespread colonization in human populations and its ability to thrive in diverse environments. However, recent trends indicate a shift, with non-albicans species increasingly isolated from clinical samples. Among these emerging pathogens, *Candida auris* stands out as a particularly alarming development. Recognized as a multidrug-resistant organism, *C. auris* has garnered significant attention from global health authorities. In 2016, both the U.S. Centers for Disease Control and Prevention (CDC) and Public Health England issued urgent alerts regarding this species, highlighting its resistance to multiple antifungal drugs and its potential to cause outbreaks in healthcare settings [2,3].

The challenges posed by *C. auris* are multifaceted and include several critical aspects that complicate its management:

*Higher morbidity and mortality rates*: Infections with *C. auris* often result in worse outcomes compared to other Candida species, particularly in vulnerable populations.

*Non-specific clinical presentation*: Its symptoms closely resemble those of other Candida infections, making early identification difficult without specialized testing.

*Difficulty in identification*: Traditional biochemical methods frequently misidentify *C. auris* as more common yeasts, such as *Candida haemulonii* or *C. duobushaemulonii*, delaying appropriate treatment [4].

*Multidrug resistance*: The species exhibits resistance to several key antifungal agents, limiting therapeutic options and necessitating advanced susceptibility testing.

## 3. Epidemiology

The incidence of candidemia and invasive candidiasis has risen steadily in recent decades, a trend driven by several converging factors. The growing population of immunocompromised patients—such as those with HIV/AIDS, cancer, or organ transplants—has expanded the pool of susceptible individuals. Additionally, the increasing use of invasive medical procedures, such as central venous catheters and surgical interventions, provides opportunities for fungal entry into sterile sites. Prolonged stays in intensive care units further exacerbate this risk as patients are exposed to multiple predisposing factors over extended periods [5]. Globally, estimates suggest that between 250,000 and 700,000 cases of candidemia and invasive candidiasis occur annually, though these figures are likely conservative. More recent data indicate an escalating burden, with *Candida albicans* now accounting for approximately 40–50% of isolates in many regions, reflecting its continued prominence [6]. However, non-albicans species—including *C. glabrata*, *C. parapsilosis*, and *C. auris*—have overtaken *C. albicans* in more than half of cases in certain settings, a shift attributed to the widespread use of antifungal prophylaxis and the resulting selective pressure on fungal populations [7].

A comprehensive systematic review conducted in 2023 provided a detailed snapshot of the global distribution of Candida species in invasive candidiasis cases. This analysis found *C. albicans* responsible for 46.3% of infections worldwide, followed by *C. glabrata* at 24.4%, *C. parapsilosis* at 8.1%, and *C. tropicalis* at 5.8%. These findings highlight a significant move away from *C. albicans* dominance and toward a more diverse array of non-albicans species [8]. In the United States, a 2021 surveillance study focusing on ICU patients with candidemia revealed similar trends, with non-albicans species predominating. Specifically, *C. glabrata* accounted for 29% of cases, and *C. parapsilosis* comprised 14%, underscoring the regional variability in species distribution and the influence of local healthcare practices [9].

### Epidemiology of Candida auris

The story of Candida auris begins with its first documented identification in 2009, when it was isolated from the ear canal of a patient in Japan—hence, its name, derived from the Latin word for ear, “auris” [10]. Subsequent retrospective analyses traced even earlier infections to South Korea in 1996, suggesting that the species may have been circulating undetected for years [11]. Since its initial discovery, *C. auris* has spread rapidly across the globe, with its incidence nearly doubling between 2019 and 2021 according to CDC data. By 2023, the CDC reported over 2377 clinical cases in the United States alone, a testament to its growing prevalence [12]. Laboratory studies have revealed that *C. auris* exhibits near-universal resistance to fluconazole, with resistance rates approaching 90–100% across isolates, though this varies by genetic clade. Resistance to amphotericin B is observed in approximately 35% of isolates, while resistance to echinocandins ranges from 5 to 10%, making it a formidable adversary in clinical settings [13].

Outbreaks of *C. auris* have been documented in multiple countries, illustrating its capacity to spread within healthcare facilities. In the United States, an initial case identified in Southern California in 2019 triggered a rapid response after it led to 182 additional cases across 17 facilities by October of that year. Enhanced hygiene measures and strict infection control protocols eventually brought this outbreak under control. Similarly, a 2015–2017 outbreak in the United Kingdom, centered in neurologic ICUs, affected 70 patients and was linked to the use of axillary temperature probes, highlighting the role of contaminated medical equipment in transmission [14]. In South Africa, *C. auris* has risen to become the third-leading cause of candidemia by 2022, accounting for 15% of cases and reflecting its growing foothold in the region [15]. Globally, the true prevalence of *C. auris* is likely underestimated due to diagnostic challenges, with particularly sharp increases noted in India (where it affects 5–10 per 100,000 ICU patients) and Latin America (where it constitutes up to 20% of candidemia cases) [16].

## 4. Risk Factors for Candidemia

### 4.1. Intensive Care

Intensive care units serve as a primary setting for candidemia cases within hospitals, largely due to the concentration of critically ill patients and the frequent use of invasive interventions. Beyond general ICUs, specialized units such as those for trauma and burn surgery also report elevated rates of infection. Specific risk factors within the ICU environment include high Acute Physiology and Chronic Health Evaluation (APACHE) scores, which indicate greater disease severity; the presence of central venous catheters, which can become colonized by fungi; and the administration of total parenteral nutrition (TPN), which provides a nutrient-rich environment conducive to fungal growth. Additional contributors include the use of broad-spectrum antibiotics, which disrupt normal microbial flora; infections with *Clostridium difficile*, which further destabilize gut microbiota; gastrointestinal perforations or anastomotic leaks, which allow fungal translocation; pancreatitis, which compromises local defenses; and hemodialysis, which involves repeated vascular access and immune suppression [17,18].

### 4.2. Immunosuppression

Patients with compromised immune systems face a significantly heightened risk of candidemia and invasive candidiasis, particularly those who have undergone hematopoietic stem cell transplantation, suffer from hematologic malignancies (e.g., leukemia or lymphoma), or have received solid organ transplants (e.g., kidney or liver). These conditions impair the body’s ability to mount an effective immune response, allowing opportunistic pathogens like Candida to proliferate unchecked [18,19]. In healthcare facilities specializing in the treatment of hematologic malignancies, non-albicans species such as *C. glabrata* and *C. krusei* often predominate, a pattern observed in a retrospective study of 635 cancer patients with candidemia spanning 1993 to 2003 [20].

### 4.3. COVID-19-Associated Candidemia

The emergence of the COVID-19 pandemic has introduced a new dimension to the epidemiology of candidemia. Severe cases of COVID-19, particularly those requiring ICU admission, have been associated with a marked increase in fungal co-infections. In a study involving 148 ICU patients, 19% developed candidemia, a finding attributed to the prolonged use of mechanical ventilation, high-dose corticosteroids, renal replacement therapy, and immunomodulatory drugs—all of which suppress immune function and create opportunities for fungal invasion. Another surveillance study reported that 25% of candidemia patients had concurrent COVID-19, further emphasizing the interplay between viral and fungal infections in critically ill populations [21].

### 4.4. Intravenous Drug Use (IVDU)

Beyond hospital settings, intravenous drug use represents a significant risk factor for community-acquired candidemia. This is particularly prevalent among younger adults aged 19–44, who may introduce Candida species directly into the bloodstream through contaminated needles or injection practices. The chronic nature of IVDU and its association with poor hygiene amplify this risk, making it a notable public health concern [20].

### 4.5. Host Factors

Individual susceptibility to Candida infections may also be influenced by genetic factors. Polymorphisms in genes encoding toll-like receptors and cytokine pathways—key components of the innate immune system—can alter the body’s ability to recognize and combat fungal pathogens, thereby increasing the likelihood of infection [22,23].

### 4.6. Risk Factors for C. auris Infection

Infections with *C. auris* are particularly associated with patients who have underlying chronic conditions and prolonged exposure to high-acuity healthcare environments, such as those requiring mechanical ventilation or long-term care. These settings provide the ideal conditions for *C. auris* to colonize and infect vulnerable individuals [23].

## 5. Pathogenesis

Candida species gain access to the bloodstream and deeper tissues through three primary mechanisms, each reflecting the opportunistic nature of these fungi:

*Gastrointestinal Tract:* As part of the normal gut microbiota, Candida species are well positioned to exploit breaches in the GI mucosa. In ICU patients or those with neutropenia, factors such as chemotherapy-induced mucosal damage, microbial overgrowth, or microperforations facilitate the translocation of fungi into the lymphatic system and subsequently the bloodstream [24].

*Intravascular Catheters:* Devices such as central venous catheters provide a direct conduit for Candida entry. Colonization can occur at the insertion site or within the catheter lumen, with TPN enhancing the risk by promoting biofilm formation—a protective matrix that shields fungi from immune responses and antifungal agents [7,17].

*Localized Focus:* Though less common, candidemia may originate from localized infections, such as urinary tract infections in the presence of obstructed urine flow, which allow fungi to disseminate hematogenously.

While colonization with Candida is a necessary precursor, additional risk factors—such as immune suppression or barrier disruption—are required for the progression to candidemia or invasive candidiasis.

## 6. Clinical Evaluation

Candidemia can seed metastatic foci in organs such as the eyes (endophthalmitis), heart (endocarditis), kidneys, joints, or others. Physicians must diligently assess for these complications:

*Ophthalmologic Examination:* Routine eye exams are recommended for all candidemia patients to detect endophthalmitis, per Infectious Diseases Society of America (IDSA) guidelines [25]. Early diagnosis prevents vision loss. In neutropenic patients, exams should be deferred until neutrophil recovery as inflammation may be minimal. Though rare, endophthalmitis incidence may be rising due to echinocandins’ limited intraocular penetration [26].

*Echocardiogram:* Persistent candidemia, prior endocarditis, or IVDU warrants an echocardiogram to rule out endocarditis. Routine imaging is unnecessary without these risk factors.

*Abdominal Imaging:* Liver or spleen abscesses may occur, necessitating imaging in patients with abdominal symptoms, elevated liver enzymes, or persistent fever. Hepatosplenic candidiasis, linked to chronic disseminated candidiasis, often presents as fever of unknown origin.

## 7. Management of Candidemia and Invasive Candidiasis

Treatment involves prompt antifungal therapy and targeted source control [27,28,29]. Central venous catheter removal should be individualized. Daily or alternate-day blood cultures monitor clearance; persistent candidemia despite therapy signals potential metastatic foci (e.g., endocarditis or abscesses).

Patients are classified as neutropenic or non-neutropenic, with treatment divided into initial and step-down phases:

*Initial Therapy:* Echinocandins (micafungin, anidulafungin, or caspofungin) are preferred; azoles are not first-line. Susceptibility testing for echinocandins and azoles is recommended [25].

*Step-Down Therapy:* For fluconazole-susceptible isolates and negative repeat blood cultures, patients may transition to oral fluconazole 5–7 days after starting treatment.

**Figure 1:**
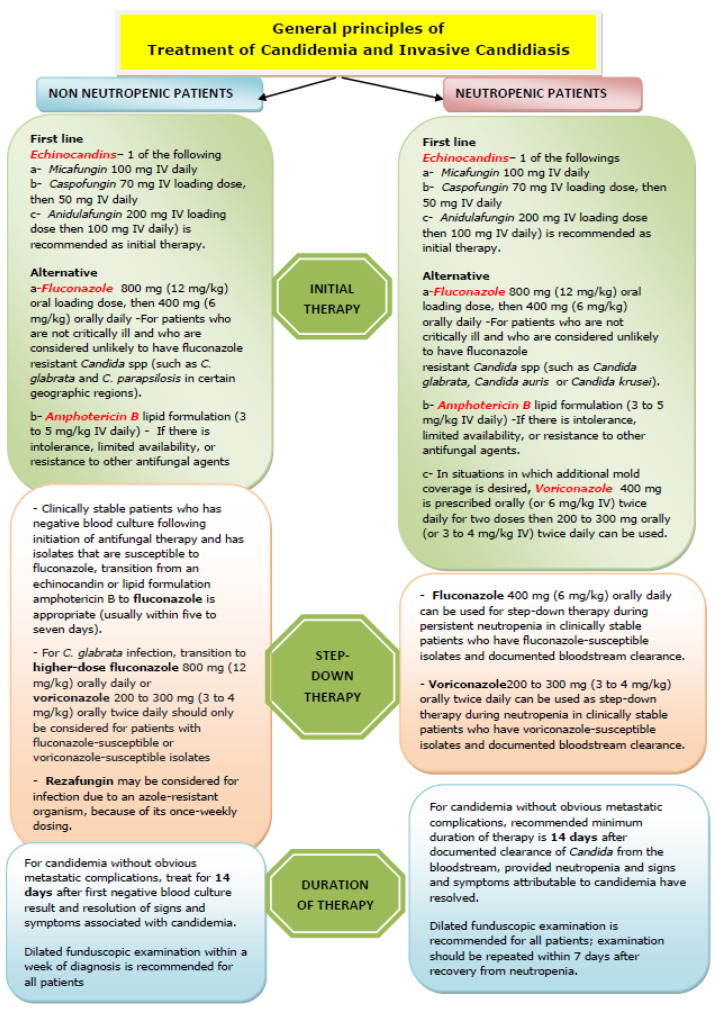
Data from Pappas P.G., Kauffman C.A., Andes D.R., et al. Clinical practice guideline for the management of candidiasis: 2016 update by the Infectious Diseases Society of America. Clin Infect Dis 2016; 62:e1 [1].

**Figure 2:**
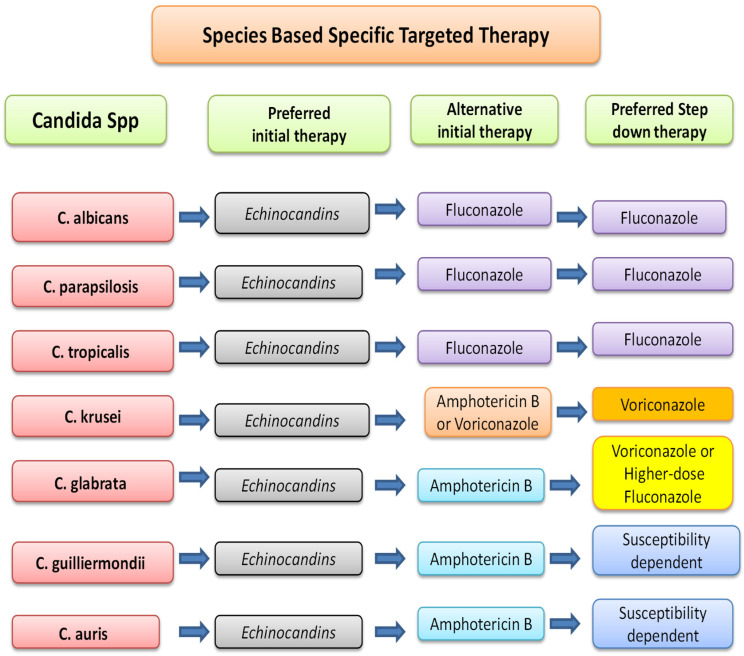
The information in this chart is based in part on Infectious Diseases Society of America (IDSA) and European Society of Clinical Microbiology and Infectious Disease (ESCMID) guidelines [25,30].

Management has been discussed in the algorithm below (Figure 1 and Figure 2).

This algorithm is based in part on the 2016 IDSA guidelines for candidiasis management [25] and incorporates ESCMID recommendations [30]. It emphasizes early antifungal initiation, source control, and tailored therapy based on patient status (neutropenic vs. non-neutropenic) and susceptibility data. For *Candida auris*, additional considerations from Section 8 (e.g., rapid resistance evolution and alternative agents like terbinafine) may apply.

## 8. Diagnosis and Management Challenges of Multidrug-Resistant *Candida auris* Infection

### 8.1. Diagnostic Challenges of C. auris

Diagnosing *Candida auris* presented initial challenges due to limitations in early commercial testing systems, which often struggled to distinguish it from species like *Candida haemulonii*, *Rhodotorulaglutinis*, or *Saccharomyces cerevisiae*. However, advancements in diagnostic technology have improved reliability, and most modern systems—including biochemical platforms, matrix-assisted laser desorption/ionization time-of-flight (MALDI-TOF) Biotyper analysis with updated *C. auris*-specific databases, and molecular sequencing of the D1/D2 domain—can now accurately identify *C. auris* [31,32]. Despite these advances, many local microbiology laboratories, particularly in resource-limited settings, lack access to these updated systems or the infrastructure to perform confirmatory tests, which may delay diagnosis and impact patient management.

### 8.2. Treatment Challenges of C. auris

*C. auris* exhibits intrinsic resistance to multiple antifungal classes, earning its classification as a multidrug-resistant species.

*Azoles:* Nearly 100% of *C. auris* isolates demonstrate high resistance to fluconazole [33,34,35,36]. Fluconazole resistance in *C. auris* depends on specific clades prevalent in different regions, with higher resistance rates observed in Clade I (South Asia) and Clade III (Africa) compared to others. Resistance to voriconazole varies (3–73%), while posaconazole, itraconazole, and isavuconazole show better activity [37].

*Amphotericin B:* Resistance in *C. auris* ranges from 13% to 35%, though accurate assessment is complicated by limitations in commercial testing methods. Systems such as Vitek and Etest often yield unreliable MICs, potentially overestimating resistance due to poor standardization compared to reference methods like broth microdilution assays recommended by the Clinical and Laboratory Standards Institute (CLSI) and European Committee on Antimicrobial Susceptibility Testing (EUCAST) [33,38,39]. These discrepancies necessitate the use of broth microdilution for precise susceptibility testing, particularly given the clinical implications of amphotericin B as a treatment option. Caution is advised when interpreting resistance rates derived from commercial assays until standardized breakpoints specific to *C. auris* are established.

*Echinocandins: C. auris* is generally susceptible (<5% resistance), though minimum inhibitory concentrations (MICs) are higher than those for *C. albicans*.

Some *C. auris* isolates exhibit elevated MICs across all three major antifungal classes (echinocandins, azoles, and polyenes) [35,40]. Specific susceptibility breakpoints for *C. auris* are not yet established; however, tentative MIC resistance thresholds—based on breakpoints for other *Candida* species—include ≥32 mcg/mL for fluconazole, ≥2 mcg/mL for amphotericin B, ≥2 mcg/mL for caspofungin, and ≥4 mcg/mL for anidulafungin and micafungin [33,41].

Resistance in *C. auris* can evolve rapidly, necessitating susceptibility testing for all initial isolates and repeat testing if infection persists despite treatment. Echinocandins remain the first-line therapy pending susceptibility results, which should be obtained as soon as possible. There is no current evidence supporting combination therapy for *C. auris* candidemia. However, dual therapy may be considered for urinary tract infections (UTIs) or central nervous system (CNS) involvement as some antifungals lack bioavailability in urine or the CNS [36].

For isolates resistant to all three major classes, susceptibility testing with flucytosine, terbinafine, and nystatin is recommended. Most *C. auris* strains remain susceptible to topical nystatin and terbinafine, suggesting potential future use of oral terbinafine for multidrug-resistant cases [36].

### 8.3. Colonization of C. auris

*C. auris* colonization is reported globally, persists longterm, and is difficult to eradicate. No reliable evidence confirms susceptibility to chlorhexidine. Nevertheless, the following strategies are recommended to prevent colonization [36]:

- Maintain high standards of care for central and peripheral lines, urinary catheters, and tracheostomies.
- Promptly remove venous cannulas at the first sign of infection.
- Adhere to strict aseptic techniques for wound care.
- Use chlorhexidine washes for skin decontamination in critically ill patients.

There is no evidence that chlorhexidine mouth gargles or topical nystatin/terbinafine at key sites (e.g., venous cannula entry points) prevent colonization or infection, though these may be considered in specific cases.

### 8.4. Screening for C. auris

Screening for *C. auris* colonization is critical to prevent healthcare-associated spread. Identifying colonized patients enables facilities to implement targeted infection prevention and control (IPC) measures. Screening decisions depend on local *C. auris* epidemiology, patient risk factors, and screening objectives. It is recommended in facilities with ongoing cases, new infections, or identified colonization. Suggested screening sites include the following:

- Groin and axilla;
- Urine;
- Nose and throat;
- Perineal swab;
- Rectal swab or stool sample.

### 8.5. Infection Prevention and Control (IPC) for C. auris

The exact mode of *C. auris* transmission in healthcare settings remains unclear but is likely multifactorial. Outbreaks in India, Pakistan, the UK, Israel, South Africa, Venezuela, and Colombia highlight its capacity to contaminate environments and equipment of colonized or infected patients [14,41]. Items in direct patient contact—such as blood pressure cuffs, stethoscopes, and pulse oximeters—are particularly prone to transmission, alongside healthcare workers’ hands. Equipment used for infected or colonized patients should not be shared unless decontamination is assured [14,41].

*C. auris* is a notifiable pathogen in the United States, with clinical cases reportable to the CDC since 2018 and screening cases reportable since 2023 [12].

## 9. Conclusions

Among fungal pathogens, *Candida* species remain the leading cause of serious invasive fungal infections. While *Candida albicans* predominates, non-*albicans* species and multidrug-resistant strains, particularly *C. auris*, are increasingly prevalent in clinical settings. Early diagnosis and the treatment of invasive candidiasis and candidemia are critical to reducing morbidity and mortality, with the timing of antifungal therapy playing a pivotal role in patient outcomes.

*C. auris* represents an emerging multidrug-resistant threat, causing fatal nosocomial infections worldwide. Its high morbidity and mortality, diagnostic challenges, and treatment difficulties due to multidrug resistance, combined with persistent colonization, underscore its status as a global health concern. Screening for colonization is the most effective strategy to curb transmission in healthcare facilities. Any *C. auris* case should be immediately reported to IPC officers and the CDC as it is a nationally notifiable pathogen.
